# Utility of a corticotropin‐releasing hormone test to differentiate pituitary‐dependent hyperadrenocorticism from cortisol‐producing adrenal tumors in dogs

**DOI:** 10.1111/jvim.16336

**Published:** 2021-12-03

**Authors:** Sachiyo Tanaka, Shuji Suzuki, Asaka Sato, Takahiro Teshima, Akihiro Mori, Toshinori Sako, Aki Tanaka, Yasushi Hara

**Affiliations:** ^1^ Faculty of Veterinary Science Laboratory of Veterinary Surgery, Nippon Veterinary and Life Science University Tokyo Japan; ^2^ Faculty of Veterinary Science Azabu University Veterinary Teaching Hospital Kanagawa Japan; ^3^ Faculty of Veterinary Science Laboratory of Veterinary Internal Medicine, Nippon Veterinary and Life Science University Tokyo Japan; ^4^ Faculty of Veterinary Science School of Veterinary Nursing and Technology, Nippon Veterinary and Life Science University Tokyo Japan; ^5^ Faculty of Veterinary Science Laboratory of Wildlife Medicine, Nippon Veterinary and Life Science University Tokyo Japan

**Keywords:** ACTH, canine, cortisol, Cushing syndrome, diagnosis

## Abstract

**Background:**

Hyperadrenocorticism (HAC) is a common endocrine disorder in dogs; however, there are no reports on the use of the corticotropin‐releasing hormone test (CRHT) to differentiate between pituitary‐dependent hyperadrenocorticism (PDH) and cortisol‐producing adrenal tumors (CPATs), both causative of HAC.

**Objectives:**

To evaluate the usefulness of CRHT as a tool to differentiate between PDH and CPAT in dogs and to determine the reference intervals for CRHT in healthy, PDH, and CPAT dogs.

**Animals:**

Dogs diagnosed with PDH (n = 21), CPAT (n = 6), and healthy beagle dogs (n = 33).

**Methods:**

This prospective study included dogs with a definitive diagnosis of PDH and CPAT and healthy beagle dogs, in which CRHT was performed, were prospectively evaluated. We investigated the correlations of CRHT (endogenous adrenocorticotropic hormone [ACTH] concentration, endogenous ACTH concentration [EAC], and poststimulation ACTH concentration [PAC]) with pituitary‐to‐brain ratio (PBR) (in PDH) and with indices of adrenal ultrasonography (smaller and larger adrenal gland dorsoventral thickness in PDH and CPAT).

**Results:**

For EAC, the area under the curve (AUC) was 0.95, with a cutoff value of 26.3 pg/mL (sensitivity: 90.62%, specificity: 87.50%). The AUC for PAC was 0.96 with a cutoff value of 54.5 pg/mL (sensitivity: 100.00%, specificity: 66.67%). The 95% reference interval for CRHT in healthy (control) dogs ranged 5.00 to 79.8 pg/mL (1.10‐17.57 pmol/L) for EAC, and 1.92 to 153.42 pg/mL (0.42‐33.78 pmol/L) for PAC. There was no significant correlation between PBR and CRHT, nor adrenal size and CRHT.

**Conclusions and Clinical Importance:**

CRHT appears to be a rapid and reliable test for differentiating PDH from CPAT in dogs.

**Abbreviations**
ACTH: adrenocorticotropic hormone
ACTHST: adrenocorticotropic hormone stimulation test
AUC: area under ROC curve
CI: confidence interval
Coe: coefficient
CPAT: cortisol‐producing adrenal tumor
CRH: corticotropin‐releasing hormone
CRHT: corticotropin‐releasing hormone test
DVTDR: dorsoventral thickness difference ratio
EAC: endogenous ACTH concentration
ECC: endogenous cortisol concentration
HAC: hyperadrenocorticism
HDDST: high‐dose dexamethasone suppression test
LAGDT: larger adrenal gland dorsoventral thickness
PAC: poststimulation ACTH concentration
PBR: pituitary‐to‐brain ratio
PCC: poststimulation cortisol concentration
PDH: pituitary‐dependent hyperadrenocorticism
SAGDT: smaller adrenal gland dorsoventral thickness
Se: sensitivity
Sp: specificity

## 1 INTRODUCTION

In small animal medicine, hyperadrenocorticism (HAC) is a common endocrine disorder, with incidence in dogs of approximately 1 to 2 cases/1000 dogs/year.^1^ Approximately 80% to 85% of HAC in dogs is caused by pituitary‐dependent hyperadrenocorticism (PDH) resulting from pituitary adrenocorticotropic hormone (ACTH)‐producing adenomas, and the remaining 15% to 20% is caused by unilateral or bilateral cortisol‐producing adrenal tumors (CPATs).^2^ Ectopic ACTH‐producing adenomas account for 5% to 15% of HACs occurring in tissues other than the pituitary gland in human medicine,^3^, ^4^, ^5^ but to our knowledge, in small animals, there is only one report of a definitive diagnosis in a dog.^3^

Considering such a background of HAC in small animal medicine, it is essential to accurately differentiate between PDH and CPAT to ensure that the appropriate treatment is implemented. The goals of treating hypercortisolism in dogs would optimally be to eliminate the source of either ACTH or autonomous cortisol excess, achieve normocortisolism, eliminate the clinical signs, reduce long‐term complications and death, and improve the quality of life.^4^ Achieving these treatment goals require definitive treatments, such as transsphenoidal hypophysectomy or radiotherapy for PDH, and adrenalectomy in the case of CPAT.^4^ PDH and CPAT are generally differentiated using a combination of imaging modalities, such as abdominal ultrasonography and MRI, and hormonal testing.^5^, ^6^ In veterinary medicine, the first choice of hormone test to differentiate PDH from CPAT is the high‐dose dexamethasone suppression test (HDDST).^5^, ^6^, ^7^, ^8^ However, HDDST requires blood sample, either 4 or 8 hours after loading, which is time‐consuming. Furthermore, in HDDST, response resistance has been reported in 25% of PDH dogs, limiting the reliability of the results.^5^ In contrast, in human medicine, several basic and clinical studies have been conducted using the human corticotropin‐releasing hormone test (CRHT) and the data obtained indicate that CRHT is useful as a diagnostic tool, especially in differentiating between PDH and CPAT, which are disorders of the hypothalamic‐pituitary‐adrenal axis.^9^, ^10^, ^11^, ^12^, ^13^, ^14^, ^15^

Corticotropin‐releasing hormone (CRH) is a hypothalamic hormone that stimulates the synthesis and secretion of ACTH in the cranial pituitary gland, and was first isolated and identified from ovines.^16^ The structure of human CRH was subsequently clarified.^17^ In small animal medicine, there have been several reports of CRHT using ovine CRH in clinical cases and experimental dogs, but to the best of our knowledge, no reports focused on the differentiation between PDH and CPAT or establishing reference intervals for CRHT in dogs.^3^, ^18^, ^19^, ^20^ Therefore, we conducted this study to evaluate the potential usefulness of CRHT as a tool to differentiate between PDH and CPAT in dogs, and examine preliminary reference intervals of CRHT in healthy, PDH, and CPAT dog cases.

## 2 MATERIALS AND METHODS

### 2.1 Dogs

Dogs diagnosed with PDH (n = 21) or CPAT (n = 6) and not treated medically or surgically for HAC were included in this study at the Department of Pituitary and Soft Tissue Surgery, Veterinary Medical Teaching Hospital, Nippon Veterinary and Life Science University between March 2006 and August 2020. Healthy beagle dogs (n = 33) were used as the control group for this study. The breeds and key characteristics of the dogs in the PDH and CPAT groups are mentioned in Table 1. The PDH group consisted of dogs with a confirmed diagnosis of pituitary corticotroph adenoma based on clinical signs, blood chemistry tests, ACTH stimulation test (ACTHST), left and right adrenal dorsoventral thickness on abdominal ultrasonography, MRI results, and histopathological examination of pituitary tissue collected during transsphenoidal surgery. The CPAT group consisted of dogs with a confirmed diagnosis of adrenal cortical adenoma based on clinical signs, blood chemistry tests, ACTHST, left and right adrenal dorsoventral thickness on abdominal ultrasonography, and histopathological examination of adrenal tissue collected at the time of adrenal tumor resection.

**TABLE 1:** Characteristics of dogs in this study including the control (33 dogs), PDH (21 dogs), and CPAT (6 dogs) groups

	Control group	PDH group	CPAT group
	n = 33	n = 21	n = 6
Breeds (n)	Beagle (33)	Mix (5) Miniature dachshunds (3) Chihuahua (3) Beagle (2) Shetland sheep dog (2) Boston terrier (1) Cavalier King Charles Spaniel (1) Maltese (1) Shiba (1) Toy poodle (1) Yorkshire terrier (1)	Shih‐tzu (2) Beagle (1) Chihuahua (1) Miniature dachshunds (1) Mix (1)
Male/female	18/15	11/10	2/4
Age (mo), median (range)	20 (9‐72)	102 (50‐172)^a^	136 (102‐177)^b^ ^,^ ^c^
BW (kg), median (range)	10.0 (8.0‐14.6)	7.6 (2.1‐26.2)	8.4 (5.8‐11.5)

*Note*: Data on age and body weight are expressed as medians (range). There were no significant differences in sex distribution between the three groups.

Abbreviations: BW, body weight; CPAT, cortisol‐producing adrenal tumor; PDH, pituitary‐dependent hyperadrenocorticism.

^a^ A significant difference between the PDH and Control group (*P* < .001).

^b^ A significant difference between the CPAT and Control group (*P* < .001).

^c^ A significant difference between the CPAT and PDH group (*P* = .001).

The control group consisted of adult beagle dogs judged healthy based on them having no evidence of systemic diseases or test results outside the reference range for physical and CBC examinations or blood chemistry tests (Table 1).

This study was approved by Animal Care and Use Committee and Ethics Committee of Veterinary Medical Teaching Hospital of Nippon Veterinary and Life Science University (approval number: 27S‐10, R2‐3), and all dogs were handled in accordance with the guidelines for laboratory animals of Animal Care and Use Committee and Ethics Committee of Nippon Veterinary and Life Science University.

### 2.2 Endocrine tests and sample collection

All PDH and CPAT dogs were food with held overnight before their visit to Veterinary Medical Teaching Hospital, Nippon Veterinary and Life Science University. After arriving at the hospital, they were placed on cage rest for 1 hour, and CRHT was performed at 10:00 am. For the control group, CRHT was performed at 10:00 am the day after overnight, once the animals had fully adapted to the environment in which they were managed at our university. All PDH and CPAT dogs underwent CRHT and ACTHST on different days under similar conditions. Thus, ACTHST was performed first, followed by CRHT. The median interval between ACTHST and CRHT was 20 days (range, 3‐43 days) in the PDH group and 19.5 days (range, 6‐42 days) in the CPAT group. None of the dogs in the PDH or CPAT groups were treated before ACTHST and CRHT were performed.

Blood samples for hormone measurements were collected, at rest, from the jugular vein. The ACTHST was performed by collecting a blood sample to measure the endogenous cortisol concentration (ECC) immediately before IV administration of synthetic ACTH (0.25 mg Cortrosyn injection, Daiichi Sankyo Co, Ltd, Tokyo, Japan) (125 μg for dogs <5 kg or 250 μg for dogs >5 kg),^21^ as well as the poststimulation cortisol concentration (PCC) at 60 minutes after administration; 17.0 to 22.0 μg/dL (469.0‐607.0 nmol/L) was defined as borderline, and PCC > 22.0 μg/dL (>607.0 nmol/L) was considered to be highly suggestive of HAC.^2^ Dogs with values above the borderline were diagnosed with HAC in conjunction with other test results (adrenal size and initial MRI).

The CRHT was performed by collecting a blood sample to measure the endogenous ACTH concentration (EAC) immediately before IV administration of 1.5 μg ovine corticotropin‐releasing factor (ovine CRF) (Peptide Institute, Inc, Osaka, Japan), as well as the poststimulation ACTH concentration (PAC) at 30 minutes after administration.^18^, ^22^, ^23^ Blood for the plasma ACTH assay was dispensed into ice‐cold tubes containing edetate disodium (EDTA‐2Na), and blood for the serum cortisol assay was dispensed into plain ice‐cold tubes. Plasma and serum were centrifuged at 1000*g* for 15 minutes at 4°C and stored at −80°C until measurements were performed. The sample storage time at −80°C was limited to 1 week in this study, with just one freeze‐thaw cycle before measuring the analytes. Serum cortisol (intra‐assay coefficients, 1.9%; interassay coefficients, 4.0%) and plasma ACTH (intra‐assay coefficients, 1.9%; interassay coefficients, 4.3%) concentrations were analyzed in each sample individually at Veterinary Medical Teaching Hospital, Nippon Veterinary and Life Science University using a chemiluminescent enzyme immunoassay (IMMULITE 1000, Siemens Healthineers, Erlangen, Germany).^24^, ^25^ The reference intervals for these assays were ECC: 1.0 to 7.8 μg/dL (27.6‐215.2 nmol/L), PCC: <20.0 μg/dL (<551.72 nmol/L), and EAC: 5.0 to 36.0 pg/mL (1.1‐7.9 pmol/L). Each dog was observed under cage rest for 2 hours after administration of ovine CRF.^19^ In the PDH and CPAT groups, the time from the onset of clinical signs to the date of CRHT and ACTHST was investigated and compared between groups.

### 2.3 Abdominal ultrasonography

All PDH and CPAT dogs underwent abdominal ultrasonography at first visit using an ultrasound system (LOGIQ S7 Expert, GE Healthcare, Tokyo, Japan; or LOGIQ 7, GE Healthcare) to measure the size of the left and right adrenal glands. Specifically, the dogs were gently restrained in the supine position without sedation. Ultrasonography was performed using a linear probe (11L‐D probe, GE Healthcare). Ultrasound images were obtained using the same settings for all dogs, except for the overall gain, which was adjusted for each dog. Color Doppler ultrasonography was performed to identify the vessels adjacent to the adrenal glands as anatomical references. In longitudinal images of each dog's left and right adrenal glands, the maximum adrenal thickness perpendicular to the long axis of the adrenal gland was measured using electronic calipers.^26^, ^27^ The dorsoventral thickness of the left and right adrenal glands obtained in each dog were compared and designated as the larger adrenal gland and the smaller adrenal gland, and recorded as larger adrenal gland dorsoventral thickness (LAGDT) and smaller adrenal gland dorsoventral thickness (SAGDT), respectively. For the symmetry of the left and right adrenal glands, the dorsoventral thickness difference ratio (DVTDR) was calculated as [DVTDR = 2 × (LAGDT − SAGDT)/(LAGDT + SAGDT)]. DVTDR ≥ 0.2 was considered as adrenal asymmetry.^28^

### 2.4 Magnetic resonance imaging

Magnetic resonance imaging was performed only in the PDH group. The cranial MRI scans were performed using a 1.5‐T superconducting MR imaging system (Visart; Toshiba, Tokyo, Japan) or a 3.0‐T superconducting MR imaging system (Signa HDxt; GE Healthcare). The MRI scans were performed under the following conditions: slice thickness of 2 mm with no slice gap, matrix of 160 × 256, and field‐of‐view of 12 cm (1.5‐T MRI), or slice thickness of 2 mm with no slice gap, matrix of 320 × 256, and field‐of‐view of 15 cm (3.0‐T MRI). The T1‐weighted images and T2‐weighted images were taken under the conditions repetition time/echo time = 410/15 ms and 4000/100 ms, respectively. The T1‐ and T2‐weighted images and gadolinium‐enhanced T1‐weighted images of the sagittal and axial images of the head were taken immediately before transsphenoidal surgery. The pituitary‐to‐brain ratio (PBR) was measured on the axial images of gadolinium‐enhanced T1‐weighted images following the method of Kooistra et al.^29^ A PBR > .31 was considered to be an enlarged pituitary gland.^29^

### 2.5 Statistical analysis

All data were subjected to the Shapiro‐Wilk test to assess normality and are expressed as median (range). The Kruskal‐Wallis test was performed to compare the variation of values within the groups (control, PDH, CPAT). Univariable linear regression was performed with dependent variables including sex distribution (male, female), age (months), body weight (kg), ECC (μg/dL), PCC (μg/dL), PAC/EAC ratio, adrenal size (LAGDT and SAGDT) (mm), the duration of time between the onset of clinical signs to CRHT (month) and PBR (in the PDH group), and independent variables of groups (control, PDH, CPAT) to evaluate the correlation. Univariable linear regression was performed with dependent variables including adrenal size (LAGDT and SAGDT) (mm) and PBR (in the PDH group) and independent valuables including EAC (pg/mL) and PAC (pg/mL) to evaluate the correlation. Sex distribution and groups were included in the models as dummy variables.

The mixed‐effect linear regression analysis was performed to evaluate the outcome variable of CRHT (EAC and PAC), the fixed effect of the groups (control, PDH, CPAT), the timing of the measurement of ACTH, and random effect of dog identities. The results are presented as coefficient (Coe), SE, *P* value, and 95% confidence interval (95% CI). The Coe shows the correlation between the outcome variable and the fixed variable. The model diagnostics were performed by evaluating the normality of the residuals, and data were log transformed for those that were not normally distributed. Post hoc analyses were performed after regression to assess the difference within the groups. For variables that were not normally distributed after log transformation, the Kruskal‐Wallis test was performed.

The area under the receiver operating characteristic (ROC) curve (AUC), cutoff value, sensitivity (Se), and specificity (Sp) were calculated by constructing ROC curves for CRHT results. The robust method was used to calculate the 95% reference interval^30^, ^31^, ^32^ and 90% CI for the lower and upper limit, and the nonparametric percentile method^33^ was used for those that had negative values for the lower limit to calculate the upper limit and lower limit for the CRHT results in each group. Statistical estimation and inference were performed using two‐tailed hypotheses and tests at the 5% significance level. The commercial statistical software Stata/IC 14.0 (StataCorp, College Station, Texas) was used to perform the Shapiro‐Wilk test, linear regression model, mixed‐effect linear regression model, margins plot, multinomial regression model, and ROC curve analysis. MedCalc Statistical Software version 19.8 (MedCalc Software Ltd, Ostend, Belgium) was used to perform 95% reference interval analysis of the CRHT results.

## 3 RESULTS

### 3.1 Adrenocorticotropic hormone stimulation test

The results of the ACTHST in the PDH and CPAT groups are shown in Tables 2, 3, 4.

**TABLE 2:** The descriptive statistics of the measured values for adrenocorticotropic hormone stimulation test (endogenous cortisol concentration, poststimulation cortisol concentration) and corticotropin‐releasing hormone stimulation test (endogenous adrenocorticotropic hormone concentration, poststimulation adrenocorticotropic hormone concentration) with median and range for control (33 dogs), PDH (21 dogs), and CPAT (6 dogs) groups

	Control group	PDH group	CPAT group	*P* value
	Median (range)	Median (range)	Median (range)	
	n = 33	n = 21	n = 6	
ACTH stimulation test				
ECC (μg/dL)	—	8.0 (2.9‐26.5)	6.7 (5.8‐41.1)	.86
PCC (μg/dL)	—	41.6^a^ (20.2‐79.2)	34.5^b^ (20.6‐65.2)	.2
CRH stimulation test				
EAC (pg/mL)	12.8 (5.0‐79.8)	110.0^f^ (10.2‐2132)	15.95^h^ (5.0‐29.4)	.15
PAC (pg/mL)	79.8^c^ (30.6‐207.0)	380.0^d^ ^,^ ^g^ (54.5‐2440)	49.9^e^ ^,^ ^i^ (18.1‐109.0)	.03

*Note*: There was no significant difference in either the EAC (*P* = .14) or PAC (*P* = 1.000) between the control and CPAT groups. The reference intervals for these assays were cortisol concentration, 1.0 to 7.8 μg/dL (27.6‐215.2 nmol/L); poststimulation plasma cortisol concentration, <20.0 μg/dL (<551.72 nmol/L); and endogenous adrenocorticotropic hormone concentration, 5.0 to 36.0 pg/mL (1.1‐7.9 pmol/L).

Abbreviations: ACTH, adrenocorticotropic hormone; CPAT, cortisol‐producing adrenal tumor; CRH, corticotropin‐releasing hormone; EAC, endogenous adrenocorticotropic hormone concentration; ECC, endogenous cortisol concentration; PAC, poststimulation adrenocorticotropic hormone concentration; PCC, poststimulation cortisol concentration; PDH, pituitary‐dependent hyperadrenocorticism.

^a^ A significant difference between PCC and ECC in the PDH group (*P* < .001).

^b^ A significant difference between PCC and ECC in the CPAT group (*P* = .03).

^c^ A significant difference between PAC and EAC in the Control group (*P* < .001).

^d^ A significant difference between PAC and EAC in the PDH group (*P* < .001).

^e^ A significant difference between PAC and EAC in the CPAT group (*P* = .02).

^f^ A significant difference in EAC between the PDH and Control groups (*P* < .001).

^g^ A significant difference in PAC between the PDH and Control groups (*P* < .001).

^h^ A significant difference in EAC between the CPAT and PDH groups (*P* < .001).

^i^ A significant difference in PAC between the CPAT and PDH groups (*P* < .001).

**TABLE 3:** Univariable linear regression for adrenocorticotropic hormone stimulation test (endogenous cortisol concentration, poststimulation cortisol concentration) and corticotropin‐releasing hormone stimulation test (poststimulation adrenocorticotropic hormone concentration) among the control (33 dogs), PDH (21 dogs), and CPAT (6 dogs) groups

Dependent variable	Independent variable	Coefficient	SE	95% confidence interval	*P* value
ECC (μg/dL)					
	PDH group	Reference			
	CPAT group	3.25	3.81	−4.56 to 11.06	.4
PCC (μg/dL)					
	PDH group	Reference			
	CPAT group	−7.99	7.09	−22.59 to 6.62	.27
PAC (μg/dL)					
	PDH group	Reference			
	Control group	−494.30	104.41	−702.05 to −285.79	<.001
	PDH group	Reference			
	CPAT group	−519.90	182.80	−886.00 to −154.71	.006

Abbreviations: CPAT, cortisol‐producing adrenal tumor; ECC, endogenous cortisol concentration; PAC, poststimulation adrenocorticotropic hormone concentration; PCC, poststimulation cortisol concentration; PDH, pituitary‐dependent hyperadrenocorticism.

**TABLE 4:** The mixed‐effect linear regression analyses for cortisol (endogenous and poststimulation cortisol concentration) (μg/dL) and adrenocorticotropic hormone (endogenous and poststimulation adrenocorticotropic hormone concentration) (pg/mL) among the control (33 dogs), PDH (21 dogs), and CPAT (6 dogs) groups and timing of pre‐ and poststimulation

Dependent outcome	Independent outcome	Coefficient	SE	95% confidence interval	*P* value
Cortisol (ECC and PCC) (μg/dL) in the PDH group	Prestimulation	Reference			
	Poststimulation	36.46	2.96	30.66‐42.26	<.001
Cortisol (ECC and PCC) (μg/dL) in the CPAT group	Prestimulation	Reference			
	Poststimulation	24.94	3.23	18.61‐31.27	<.001
ACTH (EAC and PAC) (pg/mL) in the control group	Prestimulation	Reference			
	Poststimulation	62.91	5.27	52.56‐73.25	<.001
ACTH (EAC and PAC) (pg/mL) in the PDH group	Prestimulation	Reference			
	Poststimulation	301.32	77.43	149.56‐453.09	<.001
ACTH (EAC and PAC) (pg/mL) in the CPAT group	Prestimulation	Reference			
	Poststimulation	40.38	13.40	14.10‐66.65	.003

Abbreviations: ACTH, adrenocorticotropic hormone; CPAT, cortisol‐producing adrenal tumor; EAC, endogenous adrenocorticotropic hormone concentration; ECC, endogenous cortisol concentration; PAC, poststimulation adrenocorticotropic hormone concentration; PCC, poststimulation cortisol concentration; PDH, pituitary‐dependent hyperadrenocorticism.

### 3.2 Corticotropin‐releasing hormone test

A significant increase in PAC compared to EAC was observed in the control group (*P* < .001), PDH group (*P* < .001), and CPAT group (*P* = .003). For EAC, the PDH group showed significantly higher concentrations than the control group (*P* < .001), and the CPAT group showed significantly lower concentrations than the PDH group (*P* < .001). In addition, significantly higher PAC concentrations were found in the PDH group compared to the control group (*P* < .001), and significantly lower concentrations were found in the CPAT group than in the PDH group (*P* = .006) (Tables 2, 3, 4). The post hoc analyses confirmed that there was no significant difference in PAC (*P* = .89) between the control and CPAT groups.

In the PDH group, no dog had an EAC ≤5.0 pg/mL (≤1.1 pmol/L); whereas, in the CPAT group, only two dogs (both 5.0 pg/mL) (both 1.1 pmol/L) had an EAC ≤5.0 pg/mL (≤1.1 pmol/L), and the remaining four dogs had an EAC > 5.0 pg/mL (>1.1 pmol/L), (5.1, 13.8, 25.7, 29.4 pg/mL) (1.1, 3.0, 5.7, 6.5 pmol/L). The PAC/EAC ratio was 6.01‐fold (range, 0.83‐ to 21.77‐fold) in the control group, 2.56‐fold (range, 0.58‐ to 21.65‐fold) in the PDH group, and 5.10‐fold (range, 0.98‐ to 21.8‐fold) in the CPAT group, with no significant difference between the three groups: control vs PDH groups (*P* = .1), PDH vs CPAT groups (*P* = .26), and CPAT vs control groups (*P* = .73).

**FIGURE 1:**
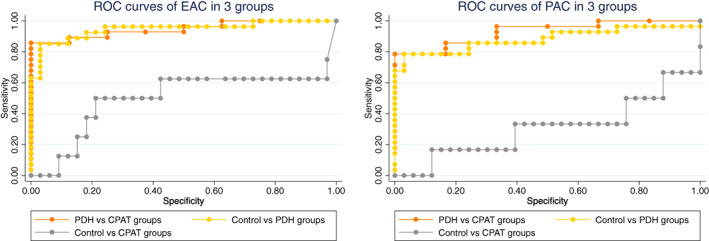
Receiver operating characteristic curve of corticotropin‐releasing hormone test in the control (33 dogs), PDH (21 dogs), and CPAT (6 dogs) groups. The area under the ROC curve for the EAC in the Control vs PDH groups was 0.95 (SE = 0.03; 95% CI, 0.90‐1.00), for the PDH vs CPAT groups was 0.95 (SE = 0.03; 95% CI, 0.89‐1.00), and in the Control vs CPAT groups was 0.500. The area under of the ROC curve for the PAC in the Control vs PDH groups was 0.92 (SE = 0.04; 95% CI, 0.84‐1.00), for the PDH vs CPAT groups was 0.96 (SE = 0.03; 95% CI, 0.89‐1.00), and in the Control vs CPAT groups was 0.308. CI, confidence interval; CPAT, cortisol‐producing adrenal tumor; EAC, endogenous adrenocorticotropic hormone concentration; PAC, poststimulation adrenocorticotropic hormone concentration; PDH, pituitary‐dependent hyperadrenocorticism; ROC, receiver operating characteristic

The ROC curves for EAC and PAC in the control, PDH, and CPAT groups are shown in Figure 1. For EAC, the AUC was 0.95 and the cutoff value was 38.3 pg/mL in the control vs PDH groups (Se, 87.10%; Sp, 96.97%). In the PDH vs CPAT groups, the AUC was 0.95 with a cutoff value of 26.3 pg/mL (Se, 90.62%; Sp, 87.50%). For PAC, in the control vs PDH groups, the AUC was 0.92 with a cutoff value of 138.0 pg/mL (Se, 83.87%; Sp, 96.97%). In the PDH vs CPAT groups, the AUC was 0.96 with a cutoff value of 54.5 pg/mL (Se, 100.00%; Sp, 66.67%).

The 95% reference interval for EAC and PAC in the three groups is shown in Table 5.

**TABLE 5:** Preliminary reference intervals for endogenous and poststimulation adrenocorticotropic hormone concentration with 95% reference interval and 90% confidence interval generated by receiver operating characteristic curve in control (33 dogs), PDH (21 dogs), and CPAT (6 dogs) groups

			Control group	PDH group	CPAT group
			n = 33	n = 21	n = 6
CRH stimulation test					
Endogenous ACTH concentration	95% reference interval	pg/mL (pmol/L)	5.0‐79.8 (1.1‐17.6)	10.2‐2132.0 (2.3‐469.5)	5.0‐29.4 (1.1‐6.5)
	90% CI for the lower limit	pg/mL (pmol/L)	NA	NA	NA
	90% CI for the upper limit	pg/mL (pmol/L)	NA	NA	NA
Poststimulation ACTH concentration	95% reference interval	pg/mL (pmol/L)	1.9‐153.4 (0.4‐33.8)	54.5‐2440.0 (12.0‐537.3)	18.1‐109.0 (4.0‐24.0)
	90% CI for the lower limit	pg/mL (pmol/L)	<25.4 (<5.6)	NA	NA
	90% CI for the upper limit	pg/mL (pmol/L)	128.8‐176.2 (28.4‐38.8)	NA	NA

Abbreviations: 90% CI, 90% confidence interval; ACTH, adrenocorticotropic hormone; CPAT, cortisol‐producing adrenal tumor; CRH, corticotropin‐releasing hormone; NA, not available; PDH, pituitary‐dependent hyperadrenocorticism.

The median time from onset of clinical signs to CRHT was 8 months (range, 3‐36 months) in the PDH group and 9.5 months (range, 3‐48 months) in the CPAT group. There was no significant correlation between CRHT result (EAC, PAC) and the duration of time between the onset of clinical signs to CRHT in the PDH group (*P* = .9 and .69, respectively) or in the CPAT group (*P* = .95 and .1, respectively).

No dogs had adverse events before CRHT or at 2 hours after‐ovine CRF administration, and no owners reported any adverse effects after their dogs returned home.

### 3.3 Abdominal ultrasonography

In the PDH group, the median LAGDT was 9.3 mm (range, 6.0‐20.0 mm) and median SAGDT was 7.9 mm (range, 5.0‐13.0 mm), with a SAGDT ≥5.0 mm in all dogs. The median DVTDR in the PDH group was 0.12 (range of 0.00‐0.54; DVTDR < 0.2 in 18/21 dogs and DVTDR ≥ 0.2 in 3/21 dogs). In the CPAT group, the median LAGDT was 17.9 mm (range, 13.9‐21.4 mm), and the median SAGDT was 6.0 mm (range, 3.4‐8.1 mm), with a SAGDT ≥5.0 mm in 3/6 dogs. The median DVTDR in the CPAT group was 1.06, ranging from 0.72 to 1.21 (DVTDR ≥ 0.2 in 6/6 dogs, all with adrenal asymmetry).

For SAGDT, there was no significant correlation with differences in the cause of HAC (PDH or CPAT) (*P* = .06), but for LAGDT, there was a significant correlation with differences in the cause of HAC (PDH or CPAT) (Coe = 0.07; SE = 0.01; 95% CI = 0.04‐0.09; *P* < .001). In the PDH and CPAT groups, there was no significant correlation between LAGDT and CRHT (EAC; *P* = .78, PAC; *P* = .41) or SAGDT and CRHT (EAC; *P* = .78, PAC; *P* = .67).

### 3.4 Magnetic resonance imaging

The median PBR in the PDH group was 0.45 (range, 0.18‐1.34). Of these, 2 of 21 dogs (PBR = 0.18, 0.25) had a PBR < 0.31^29^ and no pituitary gland enlargement. For the two dogs who did not have a pituitary enlargement on assessment of PBR, and in all dogs in the PDH group, displacement of the caudal lobe was found,^34^ and the PAC/EAC ratios were 4.60 (EAC, 67.0 pg/mL; PAC, 308.0 pg/mL) and 1.60 (EAC, 48.4 pg/mL; PAC, 77.3 pg/mL), respectively. There was no significant correlation between PBR and CRHT, or adrenal size in the PDH group.

## 4 DISCUSSION

In this study, we investigated the effect of CRH administration on plasma ACTH concentration by measuring EAC and PAC in the control, PDH, and CPAT groups. The results of this study suggest that CRHT, which is useful in human medicine, is also a valid method for distinguishing PDH from CPAT in dogs and might help guide appropriate treatment strategies for both.

Regarding the CRHT (EAC and PAC) results in this study, there was a significant difference between the control and PDH groups, and between the PDH and CPAT groups; however, no significant difference was observed between the control and CPAT groups. Therefore, CRHT might be a useful hormone test to differentiate between PDH and CPAT in cases of confirmed HAC in dogs. Indeed, our study indicated that the CRHT has a high discriminatory ability to differentiate PDH from CPAT. Besides, CRHT might be useful in differentiating pituitary ACTH‐producing microadenomas from ectopic ACTH‐producing tumors in humans^35^, ^36^ and dogs.^3^ Specifically, in human medicine, PAC/EAC increases by more than 1.5 times in many patients with PDH,^37^ while there is little change in patients with ectopic ACTH‐producing tumors,^35^, ^36^ which is a similar to in dogs with PDH^20^ and ectopic ACTH‐producing tumor.^3^ Therefore, in cases of suspected PDH without obvious enlargement of the pituitary gland, it might be useful to perform CRHT to rule out the possibility of an ectopic ACTH‐producing tumor. However, the CRHT is not intended to replace conventional screening tests for HAC in dogs. Therefore, in patients with suspected HAC, it is recommended that an endocrinological screening test such as ACTHST or low‐dose dexamethasone suppression test be performed first, followed by CRHT as a means of performing a differential diagnosis between PDH and CPAT.

The results of the 95% reference interval for CRHT in this study might be used as an example of a reference interval for CRHT in dogs. In this study, endocrine tests were performed at 10:00 am. Therefore, due to a few reports that support diurnal ACTH and cortisol concentrations,^38^, ^39^ it is recommended that CRHT be performed at a similar time of day if using these as reference values. The CRHT is advantageous as it takes a significantly shorter time to perform compared to the HDDST,^3^, ^18^, ^19^ and it is more convenient and practical in the clinical setting.

Compared to our study in which the EAC threshold was 5.0 pg/mL (1.10 pmol/L) which did not discriminate well between PDH and CPAT, previous studies indicated good discriminatory ability at this threshold.^40^ One of the possible reasons for the potential differences between our study and others might be that the PDH and CPAT dogs were under various stresses due to coming to the veterinary hospital which might have resulted in higher EAC levels.^41^, ^42^ In fact, in the report showing the above thresholds, the median EAC was 30 pg/mL (range, 6‐1250 pg/mL) in the PDH group and 5 pg/mL (range, below the limit of measurement to 5 pg/mL) in the CPAT group, which is lower than the values in the present study not only in the CPAT group but also in the PDH group. The difference in the results might also be due to the differences in the time of blood sample collection within a day^38^, ^39^ and the usage of a different version of the ACTH measurement device in the previous report.^40^ Moreover, we cannot exclude the possibility that the dogs in the CPAT group might include patients with concurrent pituitary and adrenal lesions^43^, ^44^ (imaging of the pituitary gland was not performed in the CPAT group).

In previous veterinary studies, there are reports of both abdominal ultrasonography with a threshold of 5 mm for SAGDT being able to distinguish between PDH and CPAT (Se, 100%; Sp, 96%)^28^ and of SAGDT in dogs with unilateral CPAT not being necessarily atrophic.^45^ There are inconsistencies in the literature including this study, regarding the ability of ultrasonographic adrenal indices to differentiate between PDH and CPAT.^28^, ^45^ One reason for these inconsistencies might be that in several reports, including this study, imaging of the pituitary gland was not performed in the CPAT group, which might include dogs with concurrent pituitary and adrenal lesions.^43^, ^44^

Because there is currently no canine CRF formulation available, we used ovine CRF in this study, which has been used for CRHT in dogs in several reports in the past with no reports of adverse events.^3^, ^18^, ^19^ In this study, none of the dogs had any adverse events during the 2 hours of observation after ovine CRF administration, and the owners reported no adverse effects after the dogs returned home. This suggests that the effect of a single dose of ovine CRF on the dog is minimal. However, there is still a risk that ovine CRH might be recognized as a heterologous protein in dogs, and it cannot be ruled out that repeated CRHT in the same dog might lead to an immunologic reaction to the heterologous protein.

In this study, there was no significant correlation between PBR and CRHT in PDH cases. Previous reports show that the EAC was significantly higher in PDH cases with enlarged pituitary gland size compared to cases without enlarged pituitary gland size.^29^, ^46^, ^47^ Regarding the correlation between pituitary enlargement and EAC, statistical analysis could not be performed with our results as only two cases without pituitary enlargement were included. Thus, further studies with a more significant number of PDH cases without pituitary enlargement might provide similar results to those reported in previous studies.

There are several limitations to this study. First, there was a significant difference in age between the control and case groups, and some previous veterinary reports have shown increased cortisol and ACTH concentrations in older dogs compared to younger dogs,^41^, ^48^ whereas another study reported that older dogs had significantly lower cortisol and ACTH concentrations compared to younger dogs.^49^ Therefore, a possible age‐related effect due to the significant differences in age between our groups in this study cannot be excluded. Second, we cannot rule out the possibility that the smaller sample size in the CPAT group than that in the PDH group might have lowered the statistical power of the study. Third, pituitary imaging was not performed in CPAT cases and thus, the possibility of pituitary tumors could not be ruled out in dogs classified as CPAT.

In conclusion, CRHT might be a rapid and reliable test for differentiating PDH and CPAT, which account for the majority of HAC in dogs. EAC alone was able to differentiate the patients with high sensitivity and specificity, but considering that the patients were under various stresses when they came to the hospital, evaluating PAC might be beneficial for understanding the condition of the patients. Additionally, for future research, the 95% CI for CRHT results from this study might be used as an example of a preliminary reference interval for CRHT in dogs.

## CONFLICT OF INTEREST DECLARATION

Authors declare no conflict of interest.

## OFF‐LABEL ANTIMICROBIAL DECLARATION

Authors declare no off‐label use of antimicrobials.

## INSTITUTIONAL ANIMAL CARE AND USE COMMITTEE (IACUC) OR OTHER APPROVAL DECLARATION

Approved by the Laboratory Animal Committee of Nippon Veterinary and Life Science University (approval number: S27S‐10). Also approved by the Veterinary Medicine and Veterinary Medical Teaching Hospital of Nippon Veterinary and Life Science University (approval number: R2‐3).

## HUMAN ETHICS APPROVAL DECLARATION

Authors declare human ethics approval was not needed for this study.
